# The prevalence of and factors associated with willingness to utilize HTC service among college students in China

**DOI:** 10.1186/s12889-018-5953-0

**Published:** 2018-08-22

**Authors:** Guochen Fu, Yulin Shi, Yongfu Yan, Yajie Li, Jing Han, Guosheng Li, Ruyi Lin, Yuning Wang, Zihan Fu, Qingxin Gong, Yuhang Gan, Jiaxing Wei, Junfang Wang

**Affiliations:** 1 National Demonstration Center for Experimental General Medicine Education of Hubei University of Science and Technology, Xianning, China; 2 Department of Preventive Medicine, School of Basic Medical Sciences, Hubei University of Science and Technology, No.88 Xianning Avenue, Xianning City, Hubei Province 437100 China

**Keywords:** Willingness to utilize HTC service, Influencing factors, College students, China, Logistic regression model

## Abstract

**Background:**

College students in China are emerging as one of the most vulnerable groups to contract HIV, because they are in a sexually active age group and also because of their open attitude toward sex and high risk sexual behaviors. This study aimed to explore the prevalence of willingness among college students to utilize HIV testing and counseling (HTC) service and the factors that may affect willingness, including predisposing, enabling and need factors, based on the Andersen’s behavioral model.

**Methods:**

A cross-sectional study was conducted from October 6, 2016 to December 31, 2016 in Hubei University of Science and Technology in China. After signing informed consent, college students completed a self-designed online questionnaire distributed via https://www.wjx.cn/ voluntarily, anonymously and confidentially. Pearson’s chi-square test and Logistic regression models were chosen to analyze the factors associated with willingness to utilize HTC service.

**Results:**

Out of 3314 college students in the sample, 2583 (77.9%) expressed their willingness to utilize HTC service. After adjustment, those with low levels of discrimination towards people living with HIV (PLHIV) (OR = 1.41, 95%CI:1.17–1.68), being more knowledgeable about free HTC service centers (OR = 1.44, 95%CI:1.17–1.77), having recognized the necessity to provide HTC service in the local university (OR = 2.20, 95%CI:1.73–2.80), and having a higher HIV risk perception (OR = 1.64, 95%CI:1.37–1.95) were more willing to utilize HTC service, compared with their respective counterparts.

**Conclusions:**

In order to improve their willingness to utilize HTC service and finally to achieve the goal of zero-AIDS, a comprehensive intervention measure should be taken to publicize HTC service, eliminate stigma and discrimination against PLHIV, recruit and train peer volunteers to serve in the local university, and increase self-perceived risk of HIV infection.

## Background

According to the latest figures available from the Joint United Nations Program on HIV and AIDS (UNAIDS), there were globally 36.7 million [30.8 million- 42.9 million] people living with HIV (PLHIV) at the end of 2016 and the prevalence (the percentage of HIV-infected people aged 15–49 years) has remained unchanged since 2001 and reached its highest level of 0.8% in 2015. China, compared with the global average, has a consistently lower prevalence of HIV infection (e.g., 0.06% vs. 0.8% in 2015). However, the annual number of new HIV infections in China increased sharply from 48,000 in 2011 to 115,465 in 2015. In addition, the HIV epidemic is spreading from relatively localized high-risk populations such as intravenous drug users, men who have sex with men, commercial sex workers, and former plasma donors into the general population commonly thought to be free of the HIV epidemic [1]. It is especially noted that college students in China are emerging as one of the most vulnerable groups to get HIV, because they are in a sexually active age group and also because of their open attitude toward sex (e.g., homosexuality and premarital sex) and high risk sexual behaviors (e.g., failing to use condoms correctly and consistently).

It cannot be denied that there is as yet no cure for HIV and AIDS. Fortunately, a diagnosis of HIV infection no longer means the virtual death sentence, since recent advances in bio-medical science and technology [especially the wide application of highly active antiretroviral therapy] have already turned the disease into a chronic manageable condition. In addition, UNAIDS is currently leading and inspiring the world into achieving “90–90–90” targets (90% of all PLHIV knowing their HIV status, 90% of people diagnosed with HIV infection having access to treatment and 90% of people on treatment having suppressed viral loads) by 2020 and finally “getting into zero” (zero new HIV infections, zero discrimination and zero AIDS-related deaths).

Despite clear and specific goals from UNAIDS, knowledge of HIV-positive status has remained considerably low. It is well documented that HTC service is the first and most critical step in the HIV prevention, care and treatment cascade because early detection and diagnosis of HIV infection does not only expedite early treatment and linkage to care, and it can also promote reduction in HIV-related risk behaviors. As efforts to address HTC service utilization increase, so does the need for more willingness to be tested for HIV before taking concrete action. In China, previous studies about willingness to use HTC service focused predominantly on most-at-risk populations including men who have sex with men [2–7], female sex workers [2, 3, 8], HTC service clients [2, 3, 9], and rural-to-urban migrants [10]. There have been few studies of individuals’ willingness to accept an HIV test in the general population [11–16] and three of these six studies [14–16] investigated the acceptance of HTC service through face-to-face questionnaire among college students with certain demographic characteristic such as currently pursuing a degree in medicine [14], or having had sexual experience [15], or studying in one university in metropolitan cities such as Beijing [16] and Tianjin [14] or/and in eastern coastal provinces (e.g., Zhejiang Province [15]). However, our study focused on the willingness to use HTC service from an anonymous online survey of college students in a rural comprehensive university, who had less access to HIV knowledge, prevention and resources and were more fully considered about privacy, security and confidentiality than those previously interviewed through face-to-face survey in metropolitan universities, especially medical universities. The purpose of this study is to determine the prevalence of willingness to use HTC service and the factors associated with willingness so as to identify possible interventions to ensure this type of preventive health care service is appropriately used among college students in China.

### Theoretic framework

In order to fully understand their willingness to utilize HTC service and how their willingness might be increased, the Andersen’s behavioral model [17], which assumes a sequence of predisposing, enabling and need-for-care factors contributing to an individual’s use of health care services, was chosen to serve as a conceptual framework. This model has been frequently employed in health service research for more than fifty years due to its diversity in the conceptualization and measurement of its components and also because of its flexibility to adapt to varying contexts.

Briefly, predisposing factors reflect an individual’s propensity to use health services, which includes social-demographic characteristics (e.g., gender and race) and attitudinal-belief variables. Enabling factors refer to an individual’s ability to secure services, as indicated by their own personal or family resources such as income and the availability of health services in their community of residence (e.g, price of health service). Need factors pertain to the assessment of one’s need for HTC service and comprise the subjective assessment such as self-perceived risk of HIV infection and clinical or professional evaluation of need such as risky sexual behaviors [18].

## Methods

### Design and content of the questionnaire

The structured questionnaire was developed by the Department of Preventive Medicine, School of Basic Medical Sciences of Hubei University of Science and Technology (HUST), and pilot tested with 50 students conveniently drawn from the selected population. As already mentioned above, one objective of this study is to identify factors associated with willingness to utilize HTC service among college students in China, defined as a positive response [11, 14] to the following question: “If you were offered free HTC service, would you wish to accept?” Students were then categorized into two groups: those who had expressed willingness and those who had expressed unwillingness (0 = unwillingness, 1 = willingness). Three sets of independent variables including predisposing, enabling and need factors were examined according to the Andersen’s behavioral model.

Predisposing factors in this study included gender, sexual orientation, race, grade, major, HIV-related knowledge and stigma. Knowledge about the transmitting routes and the nature of HIV infection and AIDS was measured by the 12-item scale of Yes/No/I do not know questions [11, 19], while stigma was based on the Chinese version of Zelaya’s 24-item scale of Yes/No/It depends on the situation statements [20]. The scores for knowledge and stigma scale were the total number of correct responses, with higher scores indicating greater HIV knowledge or lower levels of stigma. Given their markedly skewed distribution, the sum scores on both knowledge and stigma scale were dichotomized into high and low based on a median split.

The enabling factors included monthly living expenses, length of time in the local city, awareness of the national AIDS policy (“Four Frees and One Care” [21]), knowledge of free HIV testing sites, and recognition of the necessity to provide a free HIV test in the local university.

Need factors included HIV-related risky sexual behaviors and self-perceived risk of HIV infection [18]. HIV-related risky sexual behaviors was measured by first asking whether they had experienced or currently engaged in any form of sexual behavior. Those who picked “yes” or “refuse to answer” were then required to provide information about their risky sexual behaviors, including multiple partnerships and unsafe sex [18]. In this study, history of high-risk sexual behavior was treated as a dummy variable, which was equal to 1 if respondents had more than one sexual partner within the past 6 months or failed to use condoms consistently in every act of sexual intercourse and was 0 otherwise. Self-perceived risk of HIV infection [18] was measured by asking “what are your chances of catching HIV?” Those who answered “no possibility” were as categorized as “low perception”, all others (Not sure, Low, Moderate and High possibility) were categorized as “high perception”.

### Study sample

Participants in this cross-sectional survey were full-time undergraduate students currently registered at HUST. Most students come to this university to pursue a three-, four- or five-year degree at around the age of 18 years, which means that their ages range between 18 and 23 years. Given the fact that some university students might upgrade from junior college students or enroll earlier or later than the compulsory school attendance age, the age range in this study was then widened to be 15–26 years. This university was selected as the field of study on the basis of its convenience, diversification and comprehensiveness of majors, and its active role in preventing the rapid spread of HIV infection among college students.

### Data collection

These data were collected from October 6 to December 31, 2016. This study was approved by the academic ethics and moral supervision committee from HUST. Informed written consent was obtained from Director of Students’ Affairs Division and each class adviser for their own participation as well as on behalf of students prior to data collection, and the purpose of the study was also explained to the respondents in advance. After signing informed consent voluntarily, all the participants completed an self-designed anonymous online questionnaire distributed via https://www.wjx.cn/ and all survey responses were confidentially treated.

### Statistical analysis

Raw data collected via the website “https://www.wjx.cn/” were first transformed into excel files, then double-cleaned and analyzed independently by two authors using the Chinese version of SPSS 17.0. Any inconsistency among the two authors was discussed and then resolved with consensus of our research group members. As indicated in Tables 1 and 4, which will be discussed in detail a little later, the social-demographic variables such as gender, grade and major did not affect the willingness to utilize HTC service, thus not confounding the results. Therefore, there is no need to weight the estimates in this sample. The analysis was conducted in the three following steps. Firstly, a description of possible factors associated with the willingness to use HTC service was conducted. Secondly, variables were screened as candidate predictors on the basis of the results of the Pearson chi square test. Finally, a Binominal logistic regression model was chosen to identify variables affecting willingness to utilize HTC service. All *P* values less than 0.05 were taken as statistically significant. Odds ratio (OR) and 95% confidence interval (CI) were also calculated. All the data analysis was conducted using SPSS 17.0 software package.

**Table 1:** Bivariable analysis of factors associated with willingness to accept HIV testing among College Students (*n* = 3314)

Factors	All (*n* = 3314)		Willingness (*n* = 2583)		Unwillingness (*n* = 731)		χ2	P
	n	%	n	%	n	%		
Gender								
Female	2216	66.9	1725	66.8	491	67.2	0.04	0.84
Male	1098	33.1	858	33.2	240	32.8		
Sexual Orientation								
Heterosexual	2811	84.8	2190	84.8	621	85.0	0.01	0.91
Non-Heterosexual	503	15.2	393	15.2	110	15.0		
Race								
Non-Han	182	5.5	143	5.5	39	5.3	0.04	0.83
Han	3132	94.5	2440	94.5	692	94.7		
Grade								
Freshman	1028	31.0	791	30.6	237	32.4	1.30	0.73
Sophomore	942	28.4	733	28.4	209	28.6		
Junior	744	22.5	589	22.8	155	21.2		
Senior	600	18.1	470	18.2	130	17.8		
^b^Major								
Non-medical	1650	51.8	1240	49.7	410	59.3	20.20	< 0.001
Medical	1537	48.2	1256	50.3	281	40.7		
HIV-related knowledge (median = 10)								
Low	2019	60.9	1536	59.5	483	66.1	10.45	0.001
High	1295	39.1	1047	40.5	248	33.9		
Stigma and discrimination towards people living with HIV/AIDS (median = 18)								
High	1509	45.5	1100	42.6	409	56.0	41.03	< 0.001
Low	1805	54.5	1483	57.4	322	44.0		
Monthly living expense (Yuan RMB^a^)								
<1000	1853	55.9	1445	55.9	408	55.8	0.00	0.95
≥ 1000	1461	44.1	1138	44.1	323	44.2		
Length of time (Year)								
< 1	1241	37.4	949	36.7	292	39.9	2.50	0.11
≥1	2073	62.6	1634	63.3	439	60.1		
The awareness of the national policy on AIDS (“Four Frees and One Care”)								
No	1862	56.2	1415	54.8	447	61.1	9.39	0.002
Yes	1452	43.8	1168	45.2	284	38.9		
Knowledge of free HIV testing centers								
No	704	21.2	486	18.8	218	29.8	41.26	< 0.001
Yes	2610	78.8	2097	81.2	513	70.2		
Recognition of the necessity to provide a free HIV test in the local university								
No	375	11.3	220	8.5	155	21.2	91.38	< 0.001
Yes	2939	88.7	2363	91.5	576	78.8		
History of risky sexual behavior								
No	2844	85.8	2229	86.3	615	84.1	2.19	0.14
Yes	470	14.2	354	13.7	116	15.9		
Self-perception of HIV risk								
Low	1675	50.5	1229	47.6	446	61.0	41.12	< 0.001
High	1639	49.5	1354	52.4	285	39.0		

^a^ 1 Yuan RMB =0.1503 US dollars (October 6, 2017 rate)

^b^127 respondents were missing data for this variable

## Results

### Data quality-the effective data utilization rate

The effective data utilization rate was 87.5% (3314/3786) after excluding the 472 participants without meeting the following three criteria:①full-time undergraduate students currently registered at the local university (*n* = 407); ②the age requirement for admission to this university (15–26 years, *n* = 62); and ③answering the questionnaire beyond time restriction (from October to December 31, 2016, *n* = 3).

### Descriptive analysis

Descriptive statistics for the dependent and independent variables of this study were presented in Table 1. Out of 3314 undergraduate students in the sample, 77.9% (95% CI: 76.5–79.4%) expressed their willingness to utilize HTC service. More females (66.9%) than males (33.1%) participated in the study and the majority of respondents (94.5%) were Han. Of college students in this sample, nearly two fifths (37.4%) lived in the local city less than one year and about one third (31.0%) were freshmen. Nearly one half (48.2%) of our participants were medical students. To our surprise, 15.2% reported their sexual orientation is non-heterosexual and 55.9% spent less than one thousand Yuan on their monthly living expenses.

The total HIV knowledge score ranged from 0 to 12, with a median of 10. The frequency and percentages of correct answers for each item in knowledge scales were displayed in Table 2. No less than 90% of college students were aware that the most common routes of HIV transmission are through unprotected sex and shared needles (94.7%), blood (97.8%), and from mother to baby during pregnancy (94.1%) and that HIV is not transmitted by casual contact (90.0%). Nevertheless, knowledge regarding the nature of HIV infection was relatively low. Even approximately 40% of college students had some misconceptions regarding non-transmittable routes like mosquito bites (39.8%) and did not know about the epidemic situation of HIV infection among young students in China (43.8%). Therefore, knowledge was lacking with only 516 (15.6%) of college students answering all HIV-related knowledge questions correctly.

**Table 2:** The frequency and percentage of correct answers to HIV-related knowledge

Question	Correct responses	
	n	%
1. Can mosquito bites transmit HIV?	1996	60.2
2. Can a person be diagnosed with HIV by his appearance?	2938	88.7
3. Can causal contact with patients transmit HIV?	2984	90.0
4. Is HIV transmitted from an infected mother to her baby?	3118	94.1
5. Can receiving blood and blood products lead to HIV infection?	3241	97.8
6. Can correct and consistent use of condoms reduce the risk of HIV transmission?	2889	87.2
7. Is HIV a highly contagious, incurable disease?	2148	64.8
8. At present, is HIV spreading rapidly among young students in China, transmitted primarily through male homosexual contact and secondly through heterosexual contact?	1862	56.2
9. After taking high-risk behaviors such as needle-sharing among drug users and unprotected sexual contact, should a person immediately seek HIV voluntary counseling and test?	3138	94.7
10. Can having only one uninfected sex partner reduce the risk of HIV transmission?	2619	79.0
11. Have the rights of PLHIV been legally protected, including the rights of marriage, employment, education and medical care?	2608	78.7
12. Can use of new type of drugs such as methamphetamine increase the risk of transmitting HIV?	2460	74.2
Total correct answer about HIV-related knowledge	516	15.6

Note. Item 1–3 belong to misconceptions and the remaining items belong to correct beliefs

Generally, stigma and discrimination towards PLHIV were serious, since only 414 (12.5%) of college students were found to have positively responded to all the above-mentioned 24 situations, as shown in Table 3. More specifically, around 90% of college students were opposed to abandonment, discrimination, isolation and insult imposed on PLHIV by their family (93.0%), classmates (91.9%), partners or spouse (85.3%), and doctors (88.2%). However, a relatively lower rate of university students (70–90%) agreed that PLHIV should be allowed to work with others (68.4%) or participate in social activities (74.0%), and also disagreed that PLHIV behaved unethically, immorally or illegally (76–91%). Especially when it came to the situations such as touching the saliva (44.4%) or making friends with PLHIV (49.0%), less than one half of college students expressed willingness or fearlessness. This can be explained by the following two factors. The first possible explanation is that respondents usually provide socially acceptable rather than their true answers to sensitive questions (i.e., social desirability bias) [22], thus leading to under-reporting of value-driven stigma and individual-, family- or community-level discriminatory behavior. The second possible explanation may be related to social distance [23], which measures the degree to which college students are willing to associate with PLHIV. Along with the above-mentioned misconceptions of acquiring HIV through casual contact, the highly contagious nature of the resulting lethal AIDS significantly reduce college students’ willingness to keep an intimate contact with PLHIV.

**Table 3:** indicators and questions for measuring HIV-related stigma

Question	Correct responses	
	n	%
Fear of casual transmission and refusal of contact with PLHIV		
1. You could become infected with HIV if you are kissing PLHIV	2533	76.4
2. You could become infected with HIV if you are exposed to cough or sneeze of PLHIV	1657	50.0
3. You could become infected with HIV if you are exposed to the saliva of PLHIV	1472	44.4
4. You could become infected with HIV if you are exposed to the sweat of PLHIV	2123	64.1
5. You could become infected with HIV if you are exposed to the urine of PLHIV	1706	51.5
6. You could become infected with HIV if you are playing with PLHIV	2236	67.5
Value- and morality-related attitudes-blame, judgment and shame		
7. HIV is punishment for bad behavior	2914	87.9
8. It is women prostitutes who spread HIV	3017	91.0
9. PLHIV are promiscuous	2868	86.5
10. Only PLHIV caused by blood transfusion should be cared for and treated	2938	88.7
11. Youths might be badly influenced by PLHIV and participate in illegal activities	2479	74.8
12. Only PLHIV who stopped illegal activities should be given care and treatment	2518	76.0
Individual discriminatory behavior		
13. Doctors should treat PLHIV the same as other patients	2923	88.2
14. PLHIV should be allowed to work with others	2266	68.4
15. PLHIV should be allowed to participate in social activities	2451	74.0
16. PLHIV should be segregated	2300	69.4
17. PLHIV should be treated the same like other patients	2870	86.6
18. PLHIV but not yet showing symptoms should be allowed to continue teaching	1824	55.0
Family- or community-level discriminatory behavior		
19. PLHIV should be abandoned by his/her family	3079	92.9
20. I am willing to make friends with PLHIV	1623	49.0
21. PLHIV would be dispelled by his/her family	3082	93.0
22. PLHIV would be insulted by his/her classmates	3046	91.9
23. PLHIV would be stigmatized and discriminated	3111	93.9
24. PLHIV would be abandoned by his partner or spouse	2827	85.3
Total correct answer about HIV-related stigma	414	12.5

Since 2003, China has introduced “Four Frees and One Care” policy, including free anti-retroviral drugs for those who cannot afford to pay, testing, prevention of mother-to-child transmission, and free schooling of orphans, and care and economic assistance to the households of PLHIV [21]. Until the end of 2016, nearly three fifths (56.2%) of college students were still ignorant of the national AIDS policy (Table 1).

Table 1 illustrates that the majority of college students mentioned at least one free HIV testing site (including but not limited to the Rainbow Voluntary Organization in the local university, the hospital affiliated with local university, Centers for disease control and prevention) and also recognized the necessity to provide a free HIV test in the local university (78.8 and 88.7%, respectively).

In terms of need-for-care variables, 14.2% (470/3314) admitted the history of risky sexual behaviors, including inconsistent use of condoms (*n* = 450) or/and multiple partnerships (*n* = 287). However, more than one half (50.5%) of college students perceived themselves as having no possibility of acquiring HIV (Table 1).

### Bivariable analysis

The results of the bivariable analysis were shown in Table 1. Those who expressed greater willingness to accept a free HIV test tended to be medical students, higher levels of HIV-related knowledge, lower levels of stigma and discrimination, awareness of the “Four Frees and One Care” policy, knowledge of free HIV testing centers, recognition of the necessity to provide a free HIV test in the local university, and higher perception of the risk of HIV infection. No significant differences were reported between willingness and unwillingness in gender, race, grade, length of time, sexual orientation, monthly living expense, and history of risky sexual behavior.

### Multivariable logistic regression analysis

The stepwise multiple logistic regression model predicting willingness to utilize HTC service was shown in Table 4. When all seven significant variables were included into the logistic regression model, only four variables (i.e., HIV-related stigma, knowledge of free HIV testing centers, recognition of the necessity to provide a free HIV test in the local university and self-perceived risk of HIV infection) remained statistically significantly related to willingness to utilize HTC service, while three variables including major, HIV-related knowledge, and awareness of the “Four Frees and One Care” policy lost their statistical significance, as indicated in Table 4.

**Table 4:** Multivariable Logistic analysis of factors associated with willingness to accept HIV testing among College Students (*n* = 3014)

Factors	Wals χ2	*P* Value	OR	95% CI
Major				
0 = Non-medical, 1 = Medical	2.82	0.09	1.17	0.97–1.41
HIV-related knowledge				
0 = Low, 1 = High	0.55	0.46	1.07	0.89–1.30
Stigma and discrimination towards people living with HIV/AIDS				
0 = High,1 = Low	13.48	< 0.001	1.41	1.17–1.68
Awareness of the national policy on AIDS (“Four Frees and One Care”)				
0 = No, 1 = Yes	2.66	0.10	1.16	0.97–1.39
Knowledge of free HIV testing centers				
0 = No, 1 = Yes	12.13	< 0.001	1.44	1.17–1.77
Recognition of the necessity to provide a free HIV test in the local university				
0 = No, 1 = Yes	40.71	< 0.001	2.20	1.73–2.80
Self-perception of HIV risk				
0 = Low, High = 1	30.34	< 0.001	1.64	1.37–1.95

*OR* odds ratio, *CI* confidence interval

Among all these four significant predictors, the odds ratio (OR) was the highest for recognition of the necessity to provide a free HIV test in the local university. The college students having recognized the necessity were more likely to express their willingness to utilize HTC service (OR = 2.20, 95%CI:1.73–2.80) than those having not recognized the necessity. The odds of willingness were 1.41 times (95%CI:1.17–1.68) of respondents who had lower levels of stigma, compared to that of those with high levels of stigma. In addition, being more knowledgeable about free HIV testing centers (OR = 1.44, 95%CI:1.17–1.77) and having higher HIV risk perception (OR = 1.64, 95%CI = 1.37–1.95) were significantly associated with greater willingness to use HTC service.

## Discussions

### Main finding of this study

In our sample, only 77.9% of college students expressed their willingness to utilize HTC service, far lower than the proportion, set by UNAIDS, of 90% of all PLHIV knowing their HIV status. This underscores an urgent need to promote HTC service utilization among college students. Based on the Andersen’s behavior model of health care utilization, we identified that three enabling factors (i.e., stigma and discrimination towards PLHIV, knowledge of free HIV testing centers, recognition of the necessity to provide a free HIV test in the local university) and one need factor (perceived risk of HIV infection) were significantly associated with the willingness to use HTC service. Those with more-enabling resources (e.g. less discriminatory attitude) were more likely to express their willingness to use HTC service (OR = 1.41, 95%CI:1.17–1.68). In addition, college students with higher perception of the risk of HIV infection were more willing to utilize HTC service, when compared with their counterparts with lower perception (OR = 1.64, 95%CI:1.37–1.95).

### What is already known on this topic?

Previous studies conducted among most-at-risk populations and general populations have demonstrated that 28.2–89.0% of participants expressed the willingness to use HIV testing service [2–16]. The acceptance rate among college students (77.9%) in our study was similar to a more recent study in which being willing to accept a free HIV test accounted for 77.2% of those college students who had sexual behaviors from 11 cities in Zhejiang Province, China [15]. These inconsistencies may be mainly explained by the following two factors. The first possible explanation is time trends. With the further promotion of knowledge about HIV prevention and the “Four Frees and One Care” AIDS policy, people may be more willing to accept a free HIV test. The second possible explanation may be related to the types of HIV tests or HIV testing sites [2].

As with previous studies [11, 14, 15], higher levels of HIV-related knowledge were significantly associated with greater willingness to utilize HTC service. This finding is not surprising as it fits knowledge-attitude-belief-practice model. Those with higher levels of knowledge often gain a better insight into the possible symptoms of HIV, know more about the availability of HIV testing sites and use this information more effectively to utilize HTC service. Additionally, one fact that cannot be ignored is that only 14.2% of college students in this study answered all the twelve questions correctly, indicating an urgent need for intervention programs to dispel certain misconceptions about HIV and AIDS. However, after being adjusted by possible confounding factors, HIV-related knowledge in this study lost its statistical significance. This could be due to the fact that HIV-related knowledge was only indirectly associated with willingness to accept a free HIV test through other factors such as stigma and discrimination towards PLHIV and perceived risk of HIV infection. Therefore, a further study is needed to do path analysis to document the relationships between willingness to participate in free HIV tests, HIV-related knowledge, and other related indicators.

Consistent with previous research in which stigma and discrimination was identified as a barrier that prevents users from expressing their willingness to utilize HTC service [14], a similar result was reported in this study. Based on Grossman and Stangl’s 2013 article [24], stigma and discrimination have significantly contributed to low rates of voluntary HIV testing and HIV-positive status disclosure, late entry into HIV care, and delayed treatment. Therefore, elimination of stigma and discrimination associated with HIV and AIDS is critical to achieve a “getting to zero” future.

Consistent with previous research in which higher perception of the risk of HIV infection was significantly associated with greater willingness to use HTC service [15], a similar result was reported in this study. However, no consistent conclusion has been reached about the relationship between HIV-related risky behavior and acceptance of HIV testing. For example, Swenson et al. [25] reported that the acceptance of HIV testing in the past 3 months were less likely to have multiply sexual partner. Conversely, a more recent study conducted by Coeytaux and his colleagues [26] showed that HIV testing was positively associated with HIV-related risk behaviors (including having multiple sexual partners and inconsistent condom use) among sexually active high school students. However, no significant effect of risky sexual behavior was found on the willingness to accept a free HIV test in our study, where 14.2% of the participating students had admitted to have sexual risk-taking behavior (e.g., having multiple partners and unprotected sex). It is possible that college students in our study had the tendency to systematically underestimate their own risk of acquiring HIV in comparison to the real risk (commonly known as the optimistic bias) and treated HIV infection as a distant possibility [18], or they did not dare to admit to being at risk of HIV because they feared being stigmatized as homosexuals and prostitution. Therefore, in order to promote the adoption of protective behaviors (e.g., consistent condom use and acceptance of HIV testing after the high-risk behavior), information about the risk of HIV infection associated with exposure to sexual high-risk behaviors should be provided and the long held associations of HIV infection with homosexuals and prostitution need to be clarified, recommended by Jiang and his colleagues [15].

The concentration of willingness to utilize HTC service among college students with knowledge of free HIV testing centers and recognition of the necessity to provide a free HIV test in the local university is especially notable. These findings from this study support previous studies that have found that the price (free vs. out- of-pocket payment), availability and accessibility of health care service (e.g., a preference for home-based testing over a clinic setting). For example, Lee et al. [27] showed that a free HIV test administered at home with immediate results is the ideal choice for introducing the HIV testing among high risk populations such as men who have sex with men.

### What this study adds?

While other studies have described unwillingness to use HTC service as a barrier to effective HIV prevention, treatment, care and support, ours is the first to identify factors associated with the likelihood of willingness based on the Andersen’s behavioral model in the rural comprehensive university as well as the whole China. Our findings suggested equal use of HTC service for equal needs has not been achieved, and that respondents with unwillingness were mainly “fewer enabling resources” (e.g., without knowledge of free HIV testing centers) and “more needs” (lower perception of the risk of HIV infection). Consequently, three types of intervention aimed at reducing unwillingness to use HTC service are recommended.

1. Conduct health education: health education interventions can increase college students’ HIV-related knowledge and reduce stigma and discrimination towards PLHIV, and also provide the information about the availability of HTC service in the local university mainly provided by the the Rainbow Voluntary Organization. Accordingly, college students with increased knowledge, less discriminatory attitude towards PLHIV, and more information about free HIV testing sites and the “Four Frees and One Care” AIDS policy will be more likely to perceive the risk of HIV infection, express their willingness and finally to effectively utilize HTC service.
2. Recruit and train volunteers to serve in the local university: our results indicated that approximately 90% of college students recognized the necessity of providing a free HIV test in the local university and those with recognition were more willing to use HTC service compared with those without recognition. Therefore, further effort should be made to recruit and train volunteers to provide free HIV testing service in the local university. As already mentioned above, HTC service was actually delivered by the Rainbow Voluntary Organization in the local university, and nearly two-year of service experience also demonstrated that our peer-volunteer service model was successful in increasing the number of interested college students (especially high-risk population such as men who have sex with men) registering to accept HIV testing.
3. Promote inter-sectional cooperation on HIV prevention and control: inter-sectional collaboration and cooperation with other departments should be enhanced. To mobilize all resources and the participation of agencies, organizations, units, citizen, and communities on HIV prevention and control, it is especially important to have harmonious coordination between sectors, because there are at least seven departments (including the departments of health, propaganda, public security, labor, invalids and social affairs, the Communist Youth League, and the Women’s Federation) at the community level that are related to HIV prevention and control.

### Limitation of the study

In reporting and interpreting the outcomes of this study, we must highlight the following three limitations. First is a cross-sectional study design. One of its biggest weaknesses lies in the fact that it cannot determine the temporal order of events. Therefore, compared to a standard longitudinal study design, it has less power to establish a cause and effect relationship between the dependent variables and their relevant factors. Secondly, willingness to undergo testing does not necessarily guarantee the actual use of HTC service. Therefore, further studies are needed to clarify the circumstances under which college students will actually use HTC service. Thirdly, our findings were not fully representative of the entire university, likely because of the voluntary anonymous online survey rather than selecting the participants to complete face-to-face interview based on the random sampling strategy.

## Conclusion

Despite the target, set by UNAIDS, of 90% of all PLHIV knowing their HIV status by 2020, only 77.9% of college students expressed their willingness to accept a free HIV test until the end of 2016. Further analysis indicated that those with low levels of stigma and discrimination towards PLHIV, having recognized the necessity to provide a free HIV test in the local university, being more knowledgeable about free HIV testing centers, and having a higher HIV risk perception were more willing to accept a free HIV test, when compared with their respective control counterparts. Application of intervention strategies such as publicizing a free HIV test, eliminating stigma and discrimination against PLHIV, recruiting and training peer volunteers to serve in the local university, and increasing perceived risk of HIV infection may be effective in improving their willingness to accept a free HIV test and finally achieving the goal of zero-AIDS.

## Abbreviations

AIDS: Acquired Immune Deficiency Syndrome
CI: Confidence interval
HIV: Human immunodeficiency virus
HTC: HIV testing and counseling
HUST: Hubei University of Science and Technology
OR: Odds ratio
PLHIV: People living with HIV
SPSS: Statistical Package for the Social Sciences
UNAIDS: The Joint United Nations Program on HIV and AIDS
